# Meeting the complexity of plant nutrient metabolism with multi-omics approaches

**DOI:** 10.1093/jxb/eraa600

**Published:** 2021-03-29

**Authors:** Elmien Heyneke, Rainer Hoefgen

**Affiliations:** Max Planck Institute of Molecular Plant Physiology, D-14476 Potsdam-Golm, Germany

**Keywords:** Metabolism, multi-omics, nutrient, systems biology

## Abstract

This article comments on:

**Henriet C, Balliau T, Aime D, Le Signor C, Kreplak J, Zivy M, Gallardo K, Vernoud V**. 2021. Proteomics of developing pea seeds reveals a complex antioxidant network underlying the response to sulfur deficiency and water stress. Journal of Experimental Botany **72**, 2611–2626.

**Understanding nutrient metabolism in plants is a key challenge for understanding processes of primary and secondary metabolism and is indispensable for plant improvement in terms of yield and quality. Nutrient imbalances will lead to growth impairments and yield depressions. Plants have developed strategies to cope with such conditions within certain margins. Modern agriculture is based on supplementing soils to provide an optimal nutrient portfolio in combination with elite cultivars of crops adapted to these conditions** (Whitcomb *et al.*, 2014). **A more sustainable agriculture strives at reducing inputs. This necessitates the development of novel cultivars to fit balanced but reduced nutrient inputs**.

Physical properties such as water availability, temperature, and soil parameters all influence nutrient availability. Suboptimal conditions lead to stress, might occur in combinations, and are often disparate in time and location. Plants need to integrate all these varying abiotic inputs during the course of their life cycle. The study by Henriet *et al.* (2021) excels at investigating with a multiple ’omics (multi-omics) approach a combination of nutrient and water depletion on crop growth and yield. A combined transcriptome (Henriet *et al.*, 2019) and proteome analysis was performed in a double stress experiment during pea seed development consisting of sulfate depletion and reduction of water availability. The study resulted in the identification of stress-regulated antioxidant systems linked to sulfate response mechanisms. More than that, the study provides databases exploitable for meta studies and suggestions on how to further access the data. With that, it is one stepping stone towards an integrated multi-omics concept (Watanabe and Hoefgen, 2019).

## Premise and promise of multi-omics

The dynamic response process to sulfate deprivation and to water stress comprises successive phases. Immediate responses such as the activation of uptake systems are followed by molecular adaptations such as alterations of resource allocation, a reorganization of cellular processes including degradation of molecules or cellular structures by autophagy, followed by phenotypic alterations such as altered root growth patterns, change of flowering times, and abortion of excess sinks by, for example, selected seed abortion, and, finally, nutrient depletion-induced senescence (NuDIS). This poses the challenge of analyzing various phases, several tissues, and several stress levels to achieve a complete understanding. At the transcriptomics and metabolomics level forsulfate metabolism, for example, quite a number of studies are available. However, plants researched in the earlier studies were often exposed to extreme and often a single condition such as full sulfate starvation, and limited by technical issues such as, for example, the incompleteness of arrays. For most pertinent approaches, one should bear in mind that growth methods, though highly practical, are usually quite artificial and intentionally ‘uni-dimensional’, as they have been designed to alter only one condition in order to reduce complexity. Sulfate is highly water soluble and hence mobile in soil. In effect, the plant response to nutrient and water deprivation is complex and multi-dimensional. Targeted analyses, though fundamental, will only reveal parts of the dynamic pleiotropic responses, while multi-omics approaches will provide the integrated response of the plant at various levels as a readout (see Box 1). Targeted analyses are nonetheless indispensable to substantiate ’omics-driven hypotheses. Yet, ’omics approaches usually aim at detecting differences between states which will, for example, miss allosteric changes in enzyme activity, for which indications could be obtained by systematic flux analyses—fluxomics. To fully explore the regulatory interactions, multi-omics approaches must be adopted that quantify the stages surrounding translation, in addition to protein and mRNA abundance. A growing field is the analysis of the translatome, plant proteome, and of post-translational changes (Aroca *et al.*, 2017; Martí *et al.*, 2020; Wang and Chu, 2020). Novel, system-wide translation methods in conjunction with mRNA and protein quantification are starting to uncover the complex interplay across the ’omic scales (Kage *et al.*, 2020). Puromycin-associated nascent chain proteomics (PUNCH-P) is one of these latest techniques (Aviner *et al.*, 2013; Aviner, 2020). It leads to a snapshot of the translatome by globally labeling newly synthesized proteins that can be quantified using MS analyses, versus the more familiar ribosome profiling technique, Ribo-Seq, which arrests translation and sequences protected mRNA fragments (Choudhary *et al.*, 2020; Fremin *et al.*, 2020). By accounting for post-translational modifications by using both mRNA and translation abundance, this provides powerful data for statistical prediction of protein abundance. Ribosome profiling technologies will revolutionize our ability to understand translational control of gene expression. The outcome of this is that translation rates have significantly higher predictive power for protein abundance than transcript levels alone, as has been shown by Parkes and Niranjan (2019). Although this approach underestimates protein modification and overestimates protein degradation, it highlights the opportunities for modeling translatomics by essentially providing a flux analysis of the proteome.

Box 1. **Overview of multi-omics approaches**

The increase in potential complexity when moving from phase to phase of the canonical information flow from genome to phenotype is huge. The four-nucleotide codes of DNA and mRNA are translated into a much more complex 20 amino acid code, with primary sequence polypeptides of varying lengths that can be folded into one of an exceptionally large number of possible conformations and chemical modifications to produce a final functional protein that can have multiple isoforms for the same protein, be derived from alternative splicing, and be modified post-translationally. Furthermore, we do not completely understand the biological role of all detectable metabolites. All the above are highly plastic and can be shaped at the level of a single cell, and may span developmental time or cellular phases to form a functional part of an ecosystem together with a plethora of other organisms. Therefore, it is imperative to understand the principles by using bioinformatics tools to sensibly choose the most suitable one(s) to progress our understanding on the individual ’omics platforms and to integrate across ’omics platforms. Techniques are still in development to analyse the phenotype of cells in a high-throughput fashion, correlating changes in the genome and the proteome to cellular phenotypes; that is, cell-omics. Finally, a major challenge to fully complete the picture is represented by environmental factors which critically influence all levels. New approaches such as Mendelian randomization analysis in which variations in genes of known function (genetic markers) are used as decoys for environmental factors to be studied in association with traits are taking flight. We give credit to Mark Stitt, of Max Planck Institute of Molecular Plant Physiology, who in his many presentations shaped the idea of transcriptomics being what plants think, proteomics what they do, and metabolomics displaying the result of these processes. Illustration of vegetative Arabidopsis plant is courtesy of Frédéric Bouché (https://doi.org/10.6084/m9.figshare.7159934.v1).

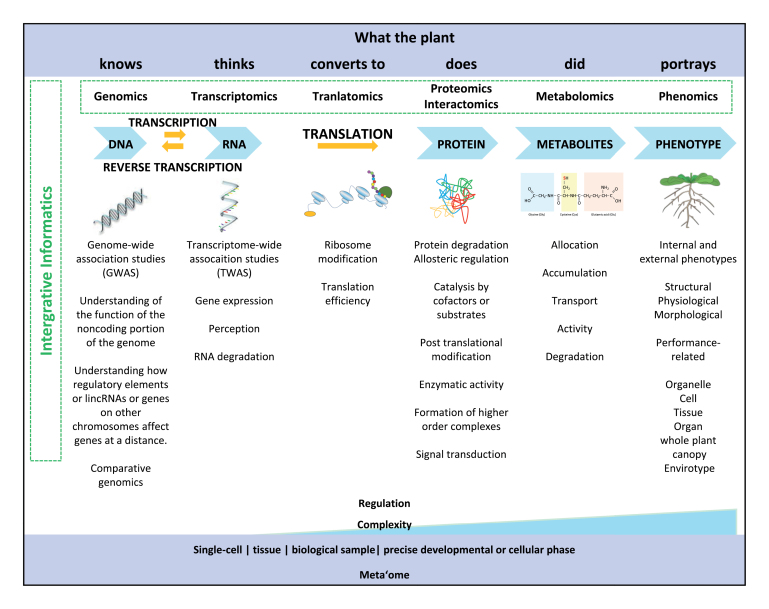

Multi-omics analyses were recently performed on simpler systems such as algae in, for example, a combined transcriptome, proteome, and metabolome investigation of the reaction of the diatom *Phaeodactylum tricornutum* to nitrogen starvation with the aim to understand the complex responses and provide tools to improve yields (Remmers *et al.*, 2018). Nitrogen starvation elevates triacylglycerol contents and carbohydrates as bioenergy precursors but with a trade-off on yield. The analysis of the complex intertwined responses of multiple pathways, initially triggered by nitrogen depletion-induced ROS (reactive oxygen species), now provides knowledge and hence possibilities for engineering improved strains for effective bi-molecule production. Another multi-omics study employing transcriptomics, metabolomics, and analysis of protein modifications and physiological parameters of the desert algae *Chlorella ohadii* which was aimed at understanding photosynthesis and performance under conditions of intensive irradiance succeeded in shedding new light on photosynthetic processes and resistance mechanism to photodamage (Treves *et al.*, 2020). More and more multi-omics studies of higher plants are being performed. Duruflé *et al.* (2020) combined phenomics, metabolomics, cell wall proteomics, and transcriptomics to explore natural variations within a population of Arabidopsis adapted to diverse environments along elevation gradients and focused on cell wall features. The authors claim that only through the combination of the multi-omics data could they identify novel genes involved in adaptation to suboptimal growth conditions. A metabolomics, proteomics, and transcriptomics study on Arabidopsis exposed to sulfate, nitrate, and phosphate starvation (Luo *et al.*, 2020) corroborated previous studies (Scheible *et al.*, 2004; Morcuende *et al.*, 2007; Bielecka *et al.*, 2015) but also provided new insights of the regulatory mechanisms involved. In general, Luo *et al.* (2020) concluded that the main transcriptomic changes related to low nitogen indicated enhancement of plant defense and immunity mechanisms, while transcriptomic changes under low sulfate indicated down-regulation of cell division and the stimulation of autophagy. Already in this study, differences from other investigations were, broadly speaking, assigned to different growth and experimental conditions. When comparing the transcriptome of a recent study by Dietzen *et al.* (2020) with that of Luo *et al.* (2020), several of the highlighted transcript changes do not coincide. On the one hand, this was described as a major challenge for comparability of multi-omics data (Watanabe and Hoefgen, 2019), but shows, on the other hand, the enormous flexibility of plant metabolism in response to its environment. Plants even adapt to the extreme microgravity conditions on the ISS space station (Kruse *et al.*, 2020). The transcriptomics, proteomics, and phosphoproteomics study in comparison with ‘earth’-grown plants identified cell wall-related processes linked to growth, ROS responses, and auxin regulation as main effects. A striking difference between the transcriptome and the proteome is mentioned in this study, and also in a recent study investigating the dynamic response of the root transcriptome and metabolome to auxin and ethylene (Hildreth *et al.*, 2020). Essentially, the transcriptome did not allow prediction of the metabolome. Kruse *et al.* (2020) claim that this is not a disadvantage, but rather indicates an as yet incomplete understanding of the regulatory landscape (see Box 1).

A common hallmark of stresses is the production of ROS. ROS are Janus-faced molecules and might act as a signal or as a threat to cell integrity (Choudhury *et al.*, 2017). The main question is how these processes are co-regulated and how general and specific responses are intertwined. Henriet *et al.* (2021) suggest a link between S starvation and drought responses, with ROS production as the prevalent result. Drought stress, as well as sulfur deprivation, causes metabolic imbalances leading to oxidative stress. ROS accumulation during sulfur deprivation stimulates peroxidation of biomolecules and reduced synthesis of sulfur-containing compounds. Considering the involvement of glutathione (GSH) in ROS detoxification pathways, it is not surprising that application of sulfate fertilizer increases the activities of the respective enzymes (Usmani *et al.*, 2020). Furthermore, sulfate application maintains the photosynthetic capacity under drought stress and promotes the photosynthetic assimilation of sulfate to produce Cys, which in turn may be used to synthesize Met or to form GSH.

## Calling for multi-omics

Plants are highly complex organisms that not only show highly specific developmental programs but also adapt in a quick and flexible manner to environmental cues. The enormous flexibility of plant metabolism, which increases from level to level, namely from DNA to phenotype, has to be considered. One example is the plethora of post-translational modifications and effects on enzyme activities at the protein level (see Box 1). To name only a few, phosphoproteomics, advanced proteomics tools such as as PUNCH-P, or protein–ligand interaction studies at high throughput scale (Luzarowski and Skirycz, 2019) might help to reveal regulatory activity changes not detectable in differential proteomics. As multi-omics studies become increasingly financially and technically attainable, and growing sets of methodologies and tools are readably available, choosing which multivariate method can generate the most insights into information locked up in complementary multi-omics data sets is of paramount importance. Just as the grand unified field theory in particle physics, the future might hold promise of one grand unified theory of ’omics: a technology that would embrace an entire life cycle and how all processes during this life cycle are shaped by perturbations and environmental factors. Acceleration of artificial intelligence from smart algorithms is the mainspring of complexomics. For this, we need a further accumulation of ’omics data, preferably multi-omics studies applied on one experimental set-up and repetitions of previous experiments with novel technologies, to broaden the database for meta-bioinformatics studies and to produce stable testable hypotheses. Given the observed discrepancies between studies, detailed descriptions of experimental procedures such as growth conditions and harvest regimes are crucial to resolve this problem and achieve comparability (Watanabe and Hoefgen, 2019). Further, a move towards analyzing organs or tissues is necessary to improve the predictive power. To this end, Henriet *et al.* (2019, 2021) provided an informative showcase as the experimental conditions for plant growth are described in detail and focus on plant seeds, eventually aiming at exploiting these data for plant breeding. Complexity might further increase when considering biotic and abiotic ecosystem effects. The integration of metadata and particular parameters such as humidity or light spectra should be used for data integration and for building prediction models. Given the vast and growing amount of data, bioinformatics will be necessary at all levels to extract processes and interconnectivities underlying metabolism and physiology, to separate the wheat from the chaff, and to achieve a systems biology understanding.

## References

[CIT0001] Aroca A , Benito JM, Gotor C, Romero LC. 2017. Persulfidation proteome reveals the regulation of protein function by hydrogen sulfide in diverse biological processes in Arabidopsis. Journal of Experimental Botany 68, 4915–4927. 28992305 10.1093/jxb/erx294 PMC5853657

[CIT0002] Aviner R . 2020. The science of puromycin: from studies of ribosome function to applications in biotechnology. Computational and Structural Biotechnology Journal 18, 1074–1083. 32435426 10.1016/j.csbj.2020.04.014 PMC7229235

[CIT0003] Aviner R , Geiger T, Elroy-Stein O. 2013. PUNCH-P for global translatome profiling. Translation 1, e27516. 26824027 10.4161/trla.27516 PMC4718054

[CIT0004] Bielecka M , Watanabe M, Morcuende R, Scheible WR, Hawkesford MJ, Hesse H, Hoefgen R. 2015. Transcriptome and metabolome analysis of plant sulfate starvation and resupply provides novel information on transcriptional regulation of metabolism associated with sulfur, nitrogen and phosphorus nutritional responses in Arabidopsis. Frontiers in Plant Science 5, 805. 25674096 10.3389/fpls.2014.00805 PMC4309162

[CIT0005] Choudhary S , Li W, Smith AD. 2020. Accurate detection of short and long active ORFs using Ribo-seq data. Bioinformatics 36, 2053–2059. 31750902 10.1093/bioinformatics/btz878 PMC7141849

[CIT0006] Choudhury FK , Rivero RM, Blumwald E, Mittler R. 2017. Reactive oxygen species, abiotic stress and stress combination. The Plant Journal 90, 856–867. 27801967 10.1111/tpj.13299

[CIT0007] Dietzen C , Koprivova A, Whitcomb SJ, Langen G, Jobe TO, Hoefgen R, Kopriva S. 2020. The transcription factor EIL1 participates in the regulation of sulfur-deficiency response. Plant Physiology 184, 2120–2136. 33060195 10.1104/pp.20.01192 PMC7723090

[CIT0008] Duruflé H , Ranocha P, Balliau T, Zivy M, Albenne C, Burlat V, Déjean S, Jamet E, Dunand C. 2020. An integrative study showing the adaptation to sub-optimal growth conditions of natural populations of *Arabidopsis thaliana*: a focus on cell wall changes. Cells 9, 2249. 10.3390/cells9102249 PMC7601860 33036444

[CIT0009] Fremin BJ , Sberro H, Bhatt AS. 2020. MetaRibo-Seq measures translation in microbiomes. Nature Communications 11, 3268. 10.1038/s41467-020-17081-z PMC7324362 32601270

[CIT0010] Henriet C , Aimé D, Térézol M, et al. 2019. Water stress combined with sulfur deficiency in pea affects yield components but mitigates the effect of deficiency on seed globulin composition. Journal of Experimental Botany 70, 4287–4304. 30855667 10.1093/jxb/erz114 PMC6698706

[CIT0011] Henriet C , Balliau T, Aime D, Le Signor C, Kreplak J, Zivy M, Gallardo K, Vernoud V. 2021. Proteomics of developing pea seeds reveals a complex antioxidant network underlying the response to sulfur deficiency and water stress. Journal of Experimental Botany 72, 2611–2626. 33558872 10.1093/jxb/eraa571

[CIT0012] Hildreth SB , Foley EE, Muday GK, Helm RF, Winkel BSJ. 2020. The dynamic response of the Arabidopsis root metabolome to auxin and ethylene is not predicted by changes in the transcriptome. Scientific Reports 10, 679. 31959762 10.1038/s41598-019-57161-9 PMC6971091

[CIT0013] Kage U , Powell JJ, Gardiner DM, Kazan K. 2020. Ribosome profiling in plants: what is not lost in translation? Journal of Experimental Botany 71, 5323–5332. 32459844 10.1093/jxb/eraa227

[CIT0014] Kruse CPS , Meyers AD, Basu P, Hutchinson S, Luesse DR, Wyatt SE. 2020. Spaceflight induces novel regulatory responses in Arabidopsis seedling as revealed by combined proteomic and transcriptomic analyses. BMC Plant Biology 20, 237. 32460700 10.1186/s12870-020-02392-6 PMC7251690

[CIT0015] Luo J , Havé M, Clément G, Tellier F, Balliau T, Launay-Avon A, Guérard F, Zivy M, Masclaux-Daubresse C. 2020. Integrating multiple omics to identify common and specific molecular changes occurring in Arabidopsis under chronic nitrate and sulfate limitations. Journal of Experimental Botany 71, 6471–6490. 32687580 10.1093/jxb/eraa337

[CIT0016] Luzarowski M , Skirycz A. 2019. Emerging strategies for the identification of protein-metabolite interactions. Journal of Experimental Botany 70, 4605–4618. 31087097 10.1093/jxb/erz228 PMC6760282

[CIT0017] Martí MC , Jiménez A, Sevilla F. 2020. Thioredoxin network in plant mitochondria: cysteine S-posttranslational modifications and stress conditions. Frontiers in Plant Science 11, 571288. 33072147 10.3389/fpls.2020.571288 PMC7539121

[CIT0018] Morcuende R , Bari R, Gibon Y, et al. 2007. Genome-wide reprogramming of metabolism and regulatory networks of Arabidopsis in response to phosphorus. Plant, Cell & Environment 30, 85–112. 10.1111/j.1365-3040.2006.01608.x 17177879

[CIT0019] Parkes GM , Niranjan M. 2019. Uncovering extensive post-translation regulation during human cell cycle progression by integrative multi-‘omics analysis. BMC Bioinformatics 20, 536. 31664894 10.1186/s12859-019-3150-5 PMC6820968

[CIT0020] Remmers IM , D’Adamo S, Martens DE, et al 2018. Orchestration of transcriptome, proteome and metabolome in the diatom *Phaeodactylum tricornutum* during nitrogen limitation. Algal Research 35, 33–49.

[CIT0021] Scheible WR , Morcuende R, Czechowski T, Fritz C, Osuna D, Palacios-Rojas N, Schindelasch D, Thimm O, Udvardi MK, Stitt M. 2004. Genome-wide reprogramming of primary and secondary metabolism, protein synthesis, cellular growth processes, and the regulatory infrastructure of Arabidopsis in response to nitrogen. Plant Physiology 136, 2483–2499. 15375205 10.1104/pp.104.047019 PMC523316

[CIT0022] Treves H , Siemiatkowska B, Luzarowska U, et al. 2020. Multi-omics reveals mechanisms of total resistance to extreme illumination of a desert alga. Nature Plants 6, 1031–1043. 32719473 10.1038/s41477-020-0729-9

[CIT0023] Usmani MM , Nawaz F, Majeed S, Shehzad MA, Ahmad KS, Akhtar G, Aqib M, Shabbir RN. 2020. Sulfate-mediated drought tolerance in maize involves regulation at physiological and biochemical levels. Scientific Reports 10, 1147. 31980688 10.1038/s41598-020-58169-2 PMC6981264

[CIT0024] Wang Y , Chu C. 2020. S-Nitrosylation control of ROS and RNS homeostasis in plants: the switching function of catalase. Molecular Plant 13, 946–948. 32445887 10.1016/j.molp.2020.05.013

[CIT0025] Watanabe M , Hoefgen R. 2019. Sulphur systems biology-making sense of omics data. Journal of Experimental Botany 70, 4155–4170. 31404467 10.1093/jxb/erz260 PMC6698701

[CIT0026] Whitcomb SJ , Heyneke E, Aarabi F, Watanabe M, Hoefgen R. 2014. Mineral nutrient depletion affects plant development and crop yield. In: Hawkesford MJ, Kopriva S, De Kok LJ, eds. Nutrient use efficiency in plants: concepts and approaches. Cham: Springer International Publishing, 205–228.

